# Development and Validation of a Machine Learning Predictive Model for Cardiac Surgery-Associated Acute Kidney Injury

**DOI:** 10.3390/jcm12031166

**Published:** 2023-02-01

**Authors:** Qian Li, Hong Lv, Yuye Chen, Jingjia Shen, Jia Shi, Chenghui Zhou

**Affiliations:** State Key Laboratory of Cardiovascular Disease, National Center for Cardiovascular Diseases, Department of Anesthesiology, Fuwai Hospital, Chinese Academy of Medical Sciences and Peking Union Medical College, 167 Beilishi Rd., Xicheng District, Beijing 100037, China

**Keywords:** cardiac surgery, acute kidney injury, machine learning, logistic regression, external validation

## Abstract

Objective: We aimed to develop and validate a predictive machine learning (ML) model for cardiac surgery associated with acute kidney injury (CSA-AKI) based on a multicenter randomized control trial (RCT) and a Medical Information Mart for Intensive Care-IV (MIMIC-IV) dataset. Methods: This was a subanalysis from a completed RCT approved by the Ethics Committee of Fuwai Hospital in Beijing, China (NCT03782350). Data from Fuwai Hospital were randomly assigned, with 80% for the training dataset and 20% for the testing dataset. The data from three other centers were used for the external validation dataset. Furthermore, the MIMIC-IV dataset was also utilized to validate the performance of the predictive model. The area under the receiver operating characteristic curve (ROC-AUC), the precision-recall curve (PR-AUC), and the calibration brier score were applied to evaluate the performance of the traditional logistic regression (LR) and eleven ML algorithms. Additionally, the Shapley Additive Explanations (SHAP) interpreter was used to explain the potential risk factors for CSA-AKI. Result: A total of 6495 eligible patients undergoing cardiopulmonary bypass (CPB) were eventually included in this study, 2416 of whom were from Fuwai Hospital (Beijing), for model development, 562 from three other cardiac centers in China, and 3517 from the MIMICIV dataset, were used, respectively, for external validation. The CatBoostClassifier algorithms outperformed other models, with excellent discrimination and calibration performance for the development, as well as the MIMIC-IV, datasets. In addition, the CatBoostClassifier achieved ROC-AUCs of 0.85, 0.67, and 0.77 and brier scores of 0.14, 0.19, and 0.16 in the testing, external, and MIMIC-IV datasets, respectively. Moreover, the utmost important risk factor, the N-terminal brain sodium peptide (NT-proBNP), was confirmed by the LASSO method in the feature section process. Notably, the SHAP explainer identified that the preoperative blood urea nitrogen level, prothrombin time, serum creatinine level, total bilirubin level, and age were positively correlated with CSA-AKI; preoperative platelets level, systolic and diastolic blood pressure, albumin level, and body weight were negatively associated with CSA-AKI. Conclusions: The CatBoostClassifier algorithms outperformed other ML models in the discrimination and calibration of CSA-AKI prediction cardiac surgery with CPB, based on a multicenter RCT and MIMIC-IV dataset. Moreover, the preoperative NT-proBNP level was confirmed to be strongly related to CSA-AKI.

## 1. Introduction

Acute kidney injury (AKI), one of the most common complications after adult cardiac surgery—with an incidence of 20% to 70%—is associated with increased short- and long-term mortality, long-term renal malfunction, and increased medical costs [1,2]. Therefore, identifying patients at high risk for AKI after cardiac surgery is fundamental for patients’ prognosis and the success of the health care system. In recent decades, cardiac surgery-associated AKI (CSA-AKI) has attracted significant attention, and researchers have been trying to establish predictive models based on potential risk factors. For the prediction of CSA-AKI, the Cleveland Clinic model, the Mehta Score, and the Simplified Renal Index system are commonly used [3,4,5]. However, these models were developed with the traditional logistic regression (LR) method, which is mainly applicable for generalized linear relationships. The machine learning (ML) method has been shown to explore the potential generalized linear and nonlinear relationships between the outcome and risk factors. Recently, several surveys have developed risk prediction models based on ML techniques [6,7,8,9,10,11,12,13]. Nevertheless, most of these model datasets were retrospectively collected from a single center, with a relatively small sample size, and have not been externally validated, resulting in limited credibility and generalizability. Hence, the evaluation and explanation of the ML models for CSA-AKI were relatively inadequate.

Using ML techniques, the “black box” could analyze large quantities of data and make decisions and predictions in the real world through a complicated algorithm. In recent decades, ML has been successfully applied to medical fields such as disease prediction [14,15] and clinical deterioration detection [16,17]. ML techniques are adept at analyzing complex information in nonlinear and highly interactive ways, exhibiting excellent performance in developing risk prediction models to assist clinicians in making decisions [18,19,20].

Therefore, this study aimed to develop and validate predictive models based on a multicenter randomized control trial (RCT) through ML and traditional LR methods. Furthermore, the Medical Information Mart for Intensive Care-IV (MIMIC-IV) dataset was also used to validate the performance of the predictive model.

## 2. Materials and Methods

### 2.1. Study Design

This was a subanalysis of a completed multicenter RCT (OPTIMAL) approved by the Fuwai Hospital Ethics Committee in Beijing, China. (NCT03782350) [21] Data about patients undergoing cardiac surgery with cardiopulmonary bypass (CPB) from 26 December 2018 to 21 April 2021 were extracted from the electronic medical records. Informed consents were obtained before enrolment in the OPTIMAL trial. This study was conducted based on the transparent reporting of a multivariable prediction model for individual prognosis or diagnosis (TRIPOD) guidelines [22].

#### Anesthetic and Surgical Procedures

All patients received general anesthesia and underwent tracheal intubation. Anesthesia was induced intravenously with midazolam (0.02–0.05 mg/kg), sufentanil (2–3 microg/kg), etomidate (0.2–0.3 mg/kg), and rocuronium (0.8–1.2 mg/kg). The maintenance of anesthesia was conducted by a continuous infusion of propofol, dexmedetomidine, and rocuronium, with an intermittent supplement of sufentanil. Sevoflurane (0.4~1.5%) was also inhaled during ventilation. Volume-controlled ventilation was maintained with a standard volume of 8 to 10 mL/kg. Arterial-line catheters were inserted into the radial arteries. Electrocardiograms, pulse oximetry, nasopharyngeal and bladder temperature, invasive arterial blood pressure, central venous pressure, blood gas analysis results, and the end-tidal carbon dioxide concentration were routinely monitored during surgery. The pulmonary artery catheters were placed as necessary.

### 2.2. Data Collection

Demographic and perioperative variables associated with postoperative AKI were gathered from the institution’s electronic medical records. Patients under 18 years of age or over 70 years of age or without outcome information were excluded. Data on patient demographics (sex, age, height, weight, New York Heart Association (NYHA) classification, systolic and diastolic pressure, left ventricular ejection fraction (LVEF), left ventricular end-diastolic diameter (LVEDD), and other baselines of the medical history) and preoperative laboratory parameters (neutrophils (NEUT), hemoglobin (HGB), platelets (PLT), alanine aminotransferase (ALT), alkaline phosphatase (AST), alkaline phosphatase (ALP), glutamyl transpeptidase (GGT), direct bilirubin (DBIL), total bilirubin (TBIL), blood creatinine (SCr), urea nitrogen (BUN), total protein (TP), albumin (ALB), N-terminal brain sodium peptide (NT-proBNP), and high-sensitivity C-reactive protein (Hs-CRP))were collected. Surgery and anesthesia-related variables, including emergency, surgery type, surgery time, aorta clamp time, and CPB time, were also collected.

### 2.3. Endpoints

The study endpoint of interest was defined as postoperative AKI, which was ascertained by Kidney Disease: Improving Global Outcomes (KDIGO) criteria based on the perioperative SCr level. [23] AKI was diagnosed when the postoperative SCr level was 1.5-fold higher than the baseline level or when an increase in SCr of 0.3 mg/dL occurred within 48 h postoperatively.

### 2.4. Model Development and Estimation

The enrolled data of Fuwai Hospital (Beijing) were randomly assigned, with 80% in the training dataset and 20% in the testing dataset. The data collected from three other cardiac centers in China were utilized for external validation (Fuwai Yunnan Cardiovascular Hospital, the First Affiliated Hospital of Wenzhou Medical University, and Fuwai Central China Cardiovascular Hospital). Moreover, the predictive models were also validated in the MIMIC-IV dataset. Development and validation datasets were imputed separately with mean values for continuous variables and frequency for categorical variables. In addition, the standard scaler data normalization technique was utilized to convert the data. Additionally, the least absolute shrinkage and selection operator (LASSO) was used to identify the variables to enter into the final model; the coefficients of variables under zero were eliminated from the model.

Data were trained on the following models: (1) LR, set as the benchmark of the traditional method, (2) support vector machine (SVM), (3) KNeighborsClassifier (KNN), (4) Naive Bayes (BAY), (5) decision tree (DT), (6) random forest (RF), (7) Gradient Boosting Classifier (GB), (8) XGBoosting Classifier (XGB), (9) Light Gradient Boosting Machine (LGBM), (10) CatBoost Classifier (CAT), (11) AdaBoostClassifier, and (12) ExtraTreeClassifier. Additionally, a grid search with five-fold cross-validation was performed on the training dataset to optimize hyperparameters.

The parameters for model discrimination (area under the receiver operating characteristic curve (ROC-AUC) and the precision-recall (PR-AUC) curve) and calibration (brier score and calibration curve) were systematically assessed. Meanwhile, the accuracy, precision, recall score, F1 score, and decision curve analysis were also assessed to evaluate the models. We selected the best-performing model based on the combination of these three metrics in the following order of priority: the highest ROC-AUC, PR-AUC, and well calibration curve. In addition, the visualization of all features was performed, along with ranked feature importance, as derived from the SHAP interpreter [22].

### 2.5. Statistical Analysis

Python programming language (Python Software Foundation, version 3.9.7 and integrated development environment JUPYTER Notebook 1.1.0) and SPSS software version 26.0 (IBM Corp., Armonk, NY, USA) were applied to our analysis. The following packages were used: data processing modules: Numpy 1.20.3, Pandas 1.3.4; data visualization module: Matplotlib 3.4.2; ML module: Scikit-Learn 0.24.2, XGBoost 1.4.2, LightGBM 3.2.1; ML interpreter: SHAP 0.39.0.

The sample size for this analysis was determined by the available data within this multicenter database. Count variables were presented in numbers and percentages, and continuous variables were presented as mean ± standard deviation (SD) or median (Q1, Q3).

## 3. Results

### Patient Characteristics

**Figure 1:**
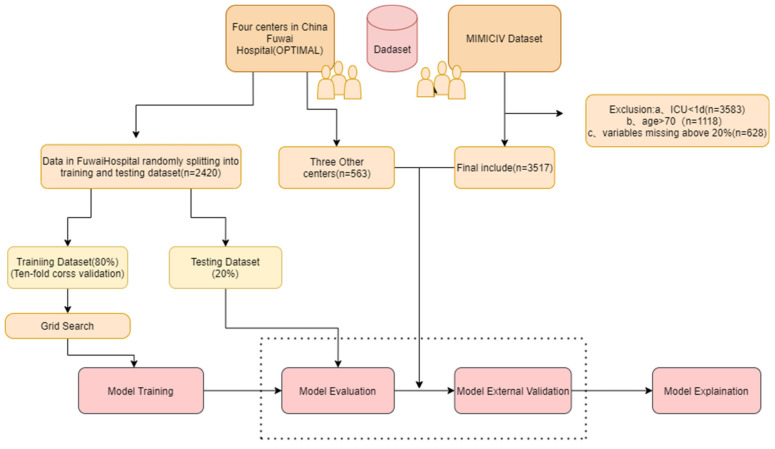
The overall review of this study.

A total of 6495 eligible patients undergoing cardiac surgery with CPB were eventually included in this study, 2416 of whom were from Fuwai Hospital (Beijing), for model development, 562 from three other cardiac centers in China, and 3517 from the MIMICIV dataset were used separately for external validation. The whole process of the study is presented in Figure 1. The patient demographics are described in Table 1. In addition, the incidence of AKI was 26.1% (630/2416) for the development set, 26.0% (146/562) for the external validation set, and 29.7% (1043/3517) for the MIMICIV dataset.

## 4. Features Selection

Ten cross-validations were utilized to select the appropriate alpha for the LASSO model. Notably, the LASSO method showed that the NT-proBNP was strongly correlated with CSA-AKI (Supplementary Figure S1). Subsequently, we excluded the NT-proBNP to determine the influence of other variables. The final enrolled variables are presented in Supplementary Figure S1.

## 5. Model Performance

**Figure 2:**
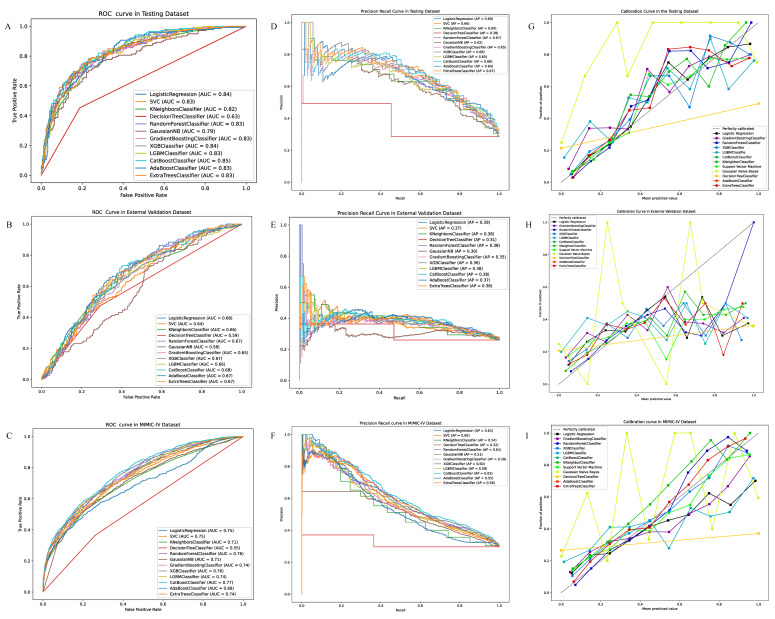
(**A**) The ROC-AUC of logistic regression and eleven machine learning algorithms in the development dataset. (**B**) The ROC-AUC of logistic regression and eleven machine learning algorithms in the validation dataset. (**C**) The ROC-AUC of logistic regression and eleven machine learning algorithms in the MIMIC-IV dataset. (**D**) The PR-AUC of logistic regression and eleven machine learning algorithms in the development dataset. (**E**) The PR-AUC of logistic regression and eleven machine learning algorithms in the validation dataset. (**F**) The PR-AUC of logistic regression and eleven machine learning algorithms in the MIMIC-IV dataset. (**G**) The calibration curve of logistic regression and eleven machine learning algorithms in the development dataset. (**H**) The PR-AUC of logistic regression and eleven machine learning algorithms in the validation dataset. (**I**) The PR-AUC of logistic regression and eleven machine learning algorithms in the validation dataset.

The discrimination of the models was presented by the ROC-AUC and PR-AUC (Figure 2). The ROC-AUC of LR achieved 0.84,0.68,0.75 in the testing, external, and MIMIC-IV validation datasets, respectively. Additionally, the best ROC-AUC were 0.85, 0.68, and 0.77 in the testing, external and MIMIC-IV datasets performed by the CAT, EX, and CAT algorithms, respectively. Furthermore, the PR-AUC of LR was 0.69, 0.39, and 0.61 in the testing, external, and MIMIC-IV validation datasets. Additionally, the best was 0.68, 0.39, and 0.63 achieved by the EX/XGB, EX, and CAT models, separately. The calibration of the models was shown by the brier score and calibration curve (Table 2 and Figure 2). The brier score of the LR model was 0.14, 0.20, and 0.19 in the testing, external, and MIMIC-IV validation datasets. In addition, the lowest score was 0.14, 0.18, and 0.16 in the testing, external, and MIMIC-IV validation dataset, achieved by the CAT, EX, and CAT models. The accuracy, precision, recall, and F1 scores are presented in Supplementary Table S1.

**Figure 3:**
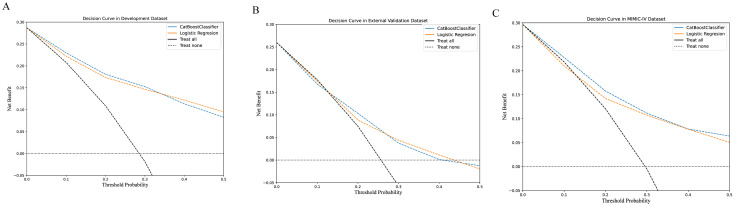
(**A**) The decision curves of logistic regression and the CatBoostClassifier models in the development dataset. (**B**) The decision curves of logistic regression and the CatBoostClassifier models in the validation dataset. (**C**) The decision curves of logistic regression and the CatBoostClassifier models in the MIMIC-IV dataset.

The decision curve between the LR and CAT models is presented in Figure 3. Moreover, the models with the perioperative surgical variables are illustrated in Supplementary Figure S2.

## 6. SHAP Interpreter for the Models

**Figure 4:**
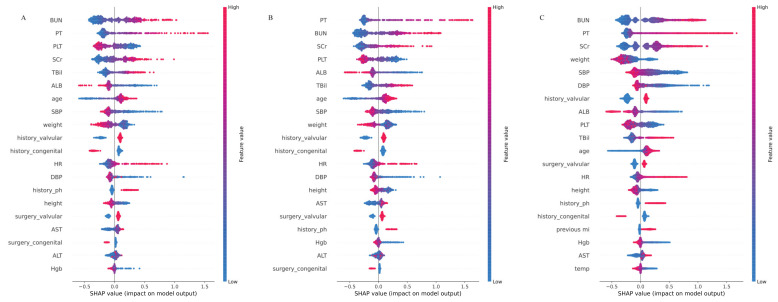
The higher the SHAP value of a feature, the higher the probability of postoperative AKI development (*X*-axis is for the SHAP values, and the *Y*-axis is for the important features. Red represents higher feature values for positive influence, and blue represents lower feature values for negative impact). (**A**) The top 20 features of the CatBoostClassifier model in the development dataset by the SHAP model explainer. (**B**) The top 20 features of the CatBoostClassifier model in the validation dataset by the SHAP model explainer. (**C**) The top 20 features of the CatBoostClassifier model in the MIMIC-IV dataset by the SHAP model explainer.

The distribution of potential risk factors was visualized and ranked by the Shapley Additive Explanations summary plots box plots using the CAT model (Figure 4). The importance matrix plot revealed that the top ten variables in the testing dataset were BUN, PT, PLT, SCr, TBil, SBP, ALB, age, height, and history of congenital conditions. In addition, the top ten features in the external validation were PT, BUN, PLT, SCr, ALB, TBil, SBP, age, weight, and history of valvular conditions. Furthermore, the top ten features in MIMC-IV were BUN, ALB, HGB, PT, SCr, DBP, weight, SBP, history of valvular conditions, and TBil.

The contribution of each feature to the overall outcome can be visualized in the plot. SHAP values for specific features exceeding zero represented a higher risk of CSA-AKI development. The models with surgical variables are presented in Supplementary Figure S2.

## 7. Discussion

In the present study, we developed and validated the predictive model of CSA-AKI with good performance using eleven ML models and the traditional LR method based on a multicenter RCT. Furthermore, external validation in the MIMIV-IV dataset also showed excellent performance. Consequently, the CAT model outperformed other ML models in regards to discrimination and calibration, showing a promising alternative for LR, with a brilliant performance in the decision curve. In addition, the top ten features in the testing, external, and MIMIC-IV datasets were comparable. Moreover, preoperative BUN, PT, SCr, TBil, and age were positively correlated with CSA-AKI; preoperative PLT, SBP, DBP, ALB, and weight were negatively correlated with CSA-AKI. These discoveries shed light on the potential for utilizing the CAT model to forecast CSA-AKI risk and guide clinical decision making in cardiac surgery.

In this study, we conducted comprehensive analyses of various ML models for CSA-AKI prediction. Moreover, our studies illustrated that the CAT model exhibited excellent discrimination and calibration qualities. The CAT algorithm, a binary recursive division technology, could yield effective outcomes with insufficient training data and computational capacity by decreasing the calculating time, overfitting the possibilities, and tuning the hyperparameter burden [24,25]. Moreover, previous studies from Tseng, P. Y., etc., repeated the positive cases five times to prevent overfitting, which may negatively impact the accuracy of the model. A grid search with five-fold cross-validation was performed in our models to avoid overfitting and obtain more accurate models [26,27]. In addition, previous studies developed predictive models for CSA-AKI without external validation, which plays an indispensable role in the models’ degree of credibility [8,10,13]. Nevertheless, we performed a good external validation based on the multicenter dataset. Of note, we utilized the MIMIC-IV dataset to validate the predictive model, with excellent performance. ML models have provided novel and convenient methods for clinicians to develop predictive models, which could greatly assist in detecting modifiable risk factors earlier and establishing standard prevention and treatment procedures for clinical practice.

Many studies have explored the independent predictors for CSA-AKI; moreover, a considerable number of studies have demonstrated that biochemistry analysis is essential for understanding the clinical events during cardiac surgery [28,29,30]. However, the available predictive models, including biochemistry biomarker predictors for CSA-AKI, are inadequate. Notably, some studies have identified novel independent risk factors, such as NT-proBNP [29] and Hs-CRP [31,32], which are associated with CSA-AKI. A recent study reported by Duchnowski, P. showed that a higher preoperative level of NT-proBNP in patients who underwent valve surgery might be related to the onset of multiple organ dysfunction syndromes (MODS), including AKI in the early postoperative period [31]. Consistent with previous studies, NT-proBNP was confirmed to be powerfully relevant to CSA-AKI in the features selection process using the LASSO method in this study. Notwithstanding, NT-proBNP, a marker of cardiac dysfunction and congestion, could be utilized to predict cardiac failure [32]. Notably, the relationship between the kidney and congestive heart failure is called “cardio-renal” syndrome, which could impact survival, the length of hospital stay, and the readmission rate [33,34]. Furthermore, patients in hemodialysis exhibit a higher NT-ProBNP, which is inclined to decrease after dialysis [33], suggesting that patients with higher NT-proBNP levels are at increased risk of developing AKI and require renal replacement treatment (RRT) [35]. Additionally, the inflammatory biomarker Hs-CRP was also identified as related to CSA-AKI by the LASSO method. The probable mechanism between inflammation and endothelial and tubular cell injury in AKI has been previously reported [36,37,38].

Consistent with previous studies, the LASSO selection method also confirmed the risk factors related to CSA-AKI, such as sex, age, LVEF, LVEDD, NYHA classification, history of hyperlipidemia, hypertension, smoking, diabetes, etc. Conspicuously, the SHAP interpreter further simplifies the prediction of the ML model, which has not been extensively used in previous surveys. In this study, we also detected that preoperative BUN, PT, SCr, TBil, and age were positively correlated with CSA-AKI; preoperative PLT, SBP, DBP, ALB, and weight were negatively correlated with CSA-AKI, which was further confirmed by external validation in the MIMIC-IV dataset. Still, the identified variables were mainly related to the function of heptanal. The relationship between the liver and the kidney is called “hepatorenal syndrome” (HRS), defined as a deterioration in kidney function occurring in severe chronic liver disease [39,40,41]. HRS could be activated by dramatically reducing the effective circulating volume and the sprout of the vasoactive endogenous system [42]. Moreover, systemic inflammation, cirrhotic cardiomyopathy, hepato-adrenal syndrome, choleric nephropathy, and intra-abdominal hypertension could further exacerbate this process [43,44]. Additional research is needed to explore the potential underlying mechanisms.

There are also some limitations to our study. First, the present study did not analyze the intraoperative fluid balance, which has been shown to play an important role in CSA-AKI. Second, owing to the lower incidence of stage 2 and 3 AKI in our study, we did not analyze the data by the stage of AKI; more research is urgently needed to establish all-stage AKI predictive models. Third, further prospective validation is required before our models are affirmatively applied to other populations, institutions, and regions. Fourth, although the sample size in this study for the ML prediction of AKI event rate/numbers is relative enough, we could not rule out that a larger population may result in better prediction performance. Fifth, some unknown potential confounding factors for CSA-AKI were not included as the features, which may result in selection bias. Finally, the development and validation of the ML models are based on retrospective datasets. Prospective validation is required before usage in clinical settings.

## 8. Conclusions

We have developed and validated a predictive model for CSA-AKI based on a multicenter RCT and an MIMIC-IV dataset. It suggested that the CatBoostClassifier algorithms outperform other ML models for both discrimination and calibration. Moreover, in this study, NT-proBNP was confirmed to be strongly related to CSA-AKI.

## Figures and Tables

**Table 1:** Demographics of development and ex-validation dataset.

	Development Dataset (*n* = 2416)	Ex-Validation Dataset (*n* = 562)	MIMIC-IV Dataset (*n* = 3517)
Sex (*n*, %)			
Male	1517 (62.8%)	322 (57.3%)	2676 (76.1%)
Female	899 (37.2%)	240 (42.7%)	841 (30.5%)
Age (y), median (Q1, Q3)	54.7 (45.8, 62.1)	56.3 (48.7, 64)	61.6 (55.3, 66)
BMI (kg/m^2^), mean ± SD	24.1 (23.1, 25.4)	23.2 (22.3, 24.9)	29.8 (27.3, 32.5)
Medical history (*n*, %)			
Diabetes	259 (10.7%)	27 (4.8%)	1227 (34.9%)
CHD	663 (27.4%)	99 (17.6%)	1997 (56.8%)
Valvular disease	1721 (71.2%)	352 (62.6%)	1071 (30.5%)
Congenital heart disease	413 (17.1%)	62 (11.0%)	31 (0.9%)
PVD	392 (16.2%)	33 (5.9%)	493 (14%)
Previous myocardial injury	392 (16.2%)	11 (5.9%)	493 (14.0%)
Hyperlipidaemia	872 (36.1%)	115 (20.5%)	2426 (69.0%)
Hypertension	802 (33.2%)	219 (39.0%)	2350 (66.8%)
COPD	4 (0.2%)	4 (0.7%)	682 (19.4%)
CKD	5 (0.2%)	6 (1.1%)	397 (11.3%)
Infective endocarditis	18 (0.7%)	12 (2.1%)	163 (4.6%)
Non-invasive tests suggesting carotid artery stenosis >79% or Stroke	109 (4.5%)	51 (9.1%)	288 (8.2%)
Vital signs			
Body temperature, °C	36.4 (36.2, 36.5)	36.5 (36.3, 36.7)	36.5 (36.1, 36.8)
Heart rate, bpm/min	77 (68, 86)	74.5 (66, 83)	80 (74, 87)
SD (mm Hg)	53 (49, 58)	45 (42, 50)	52 (48, 55)
Laboratory results			
WBC, ×10 L	6.1 (5.2, 7.2)	6.2 (5.2, 7.7)	7.6 (6.1, 9.4)
Hemoglobin/dL	127 (138, 149)	133 (121, 144)	131 (118, 144)
Platelets, ×10/L	200 (167, 240)	199.5 (164, 243.2)	211 (173, 252)
AST (U/L)	25 (21, 31)	21 (17, 28)	24 (19, 30.8)
ALT (U/L)	19 (13, 29)	19 (13, 31)	25 (18, 32)
ALP (U/L)	65 (54, 78)	76.4 (61, 83.3)	71 (57, 84)
Total bilirubin, (μmol/L)	11.9 (8.8, 16.2)	12 (8.1, 16)	8.6 (6.8, 12.0)
Baseline creatinine, (μmol/L)	82 (72, 93.2)	71 (60, 83)	88.4 (70.7, 103.5)
BUN (mmol/L)	6 (4.9, 7.4)	5.8 (4.6, 7.1)	6.1 (5.0, 7.9)
PT*s	13.1 (12.7, 13.7)	12.1 (11.0, 13.5)	12.5 (11.4, 12.9)
ALB (mg/dL)	39.8 (37.7, 41.9)	40.3 (38.2, 42.9)	41.1 (39.0, 44.0)
Surgical information			
Surgery type, *n* (%)			
Valvular	1517 (62.8%)	386 (68.7%)	1071 (30.5%)
CABG	719 (29.8%)	105 (18.7%)	1997 (56.8%)
Congenital	342 (14.2%)	60 (10.7%)	31 (0.9%)
AKI	630 (26.1%)	146 (26.0%)	1043(29.7%)

**Table 2:** The ROC-AUC and brier loss score for the development, validation and MIMI-IV datasets.

Classifier	ROC-AUC			Brier Loss		
	Development	Ex-Validation	MIMIC-IV	Development	Ex-Validation	MIMIC-IV
Logistic Regression	0.8355	0.6775	0.7450	0.1411	0.2016	0.1873
Support Vector Machine	0.8269	0.6415	0.7214	0.1454	0.2131	0.1738
KNeighborsClassifier	0.8222	0.6581	0.6942	0.1590	0.1872	0.1900
DecisionTreeClassifier	0.5993	0.5947	0.5420	0.3079	0.3434	0.3651
RandomForestClassifier	0.8295	0.6691	0.7317	0.1587	0.1829	0.1750
GaussianNB	0.7916	0.5817	0.6814	0.2476	0.2782	0.2603
GradientBoostingClassifier	0.8224	0.6491	0.6860	0.1565	0.2225	0.1846
XGBClassifier	0.8384	0.6663	0.7167	0.1446	0.2006	0.1718
LGBMClassifier	0.8333	0.6606	0.6992	0.1746	0.2432	0.2136
CatBoostClassifier	0.8455	0.6706	0.7429	0.1451	0.1920	0.1657
AdaBoostClassifier	0.8302	0.6722	0.6518	0.2355	0.2385	0.2333
ExtraTreeClassifier	0.8327	0.6799	0.7354	0.1546	0.1807	0.1771

## Data Availability

Data available on request due to restrictions privacy. The data presented in this study are available on request from the corresponding author. The data are not publicly available due to privacy.

## Supplementary Materials

The following supporting information can be downloaded at: https://www.mdpi.com/article/10.3390/jcm12031166/s1, Figure S1: (A) Feature variable selection using least absolute shrinkage and selection operator (LASSO) regression in the development cohort. The Y-axis is the features coefficient, and the X-axis is the alpha value. The feature coefficients of nearly zero were excluded. (B) LASSO methods excluded features without blue plots. (C) Feature variables without NT-pro BNP selection using least absolute shrinkage and selection operator (LASSO) regression in the development cohort. The Y-axis is the features coefficient, and the X-axis is the alpha value. The features coefficient of nearly zero were excluded. (D) LASSO methods included features with blue plots. Figure S2: (A) The AUC of the machine learning models with surgical variables in the testing dataset. (B) The AUC of the machine learning models with surgical variables in the validation dataset. (C) The precision recall curve of the machine learning models with surgical variables in the testing dataset. (D) The precision recall curve in the machine learning models with surgical variables in the validation dataset. (E) The calibration curve of the machine learning models with surgical variables in the testing dataset. (F) The calibration curve in the machine learning models with surgical variables in the validation dataset. Table S1: The accuracy, precision, recall, F1 score, and log loss in the development and external validation datasets
