# Supplementary material for: Development and Validation of a Machine Learning Predictive Model for Cardiac Surgery-Associated Acute Kidney Injury
Source: J Clin Med. 2023 Feb 1;12(3):1166. doi: 10.3390/jcm12031166 (PMC9917969; doi:10.3390/jcm12031166)
Supplement: Supplementary file 1 [file jcm-12-01166-s001.zip › jcm-2151696-supplementary.pdf]

Supplementary

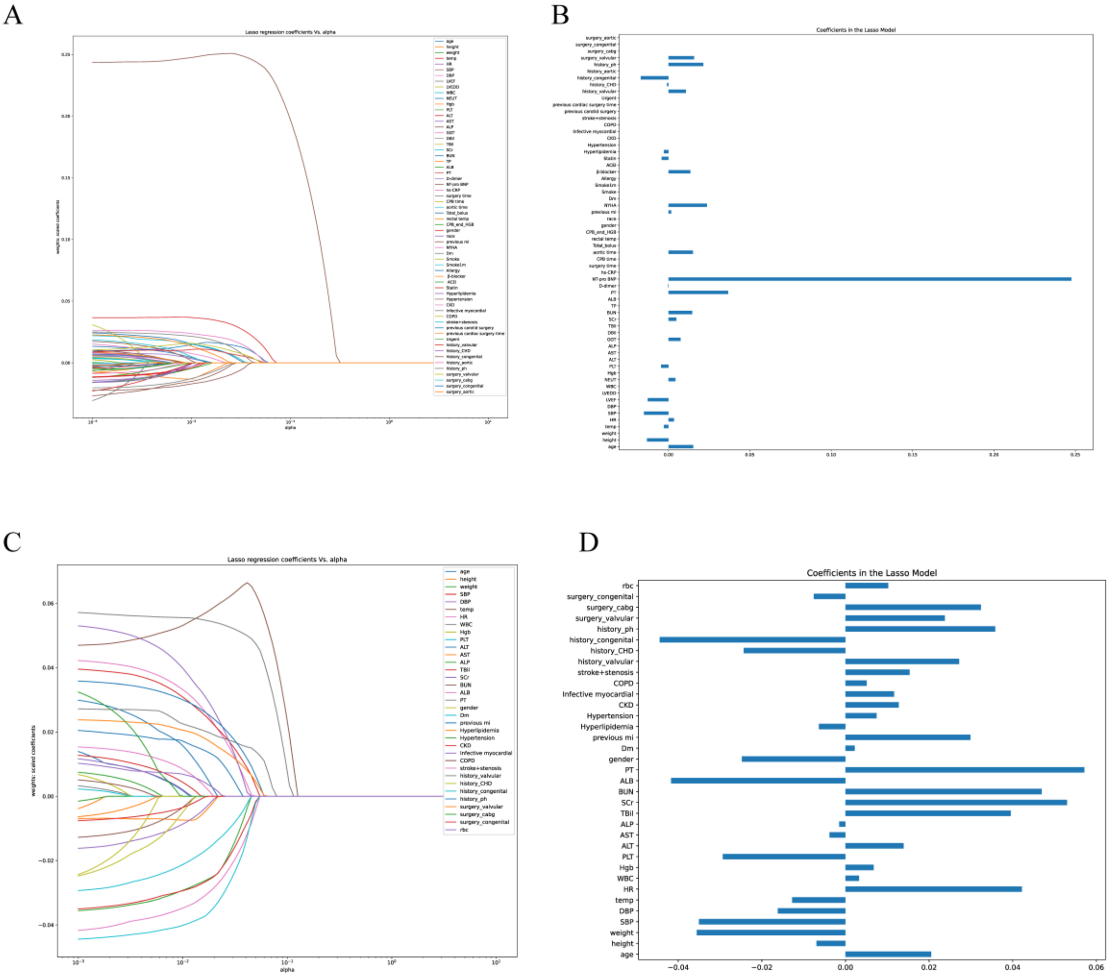

Figure S1:(A) Feature variable selection using least absolute shrinkage and selection operator (LASSO) regression in the development cohort. The Y-axis is the features coefficient, and the X-axis is the alpha value. The features coefficient of nearly zero were excluded.

(B) LASSO methods excluded features without blue plots.

(C) Feature variables without NT-pro BNP selection using least absolute shrinkage and selection operator (LASSO) regression in the development cohort. The Y-axis is the features coefficient, and the X-axis is the alpha value. The features coefficient of nearly zero were excluded.

(D) LASSO methods included features with blue plots.

Abbreviation:

BMI: Body Mass Index; Race-1: Han-Chinese; Race-2: Chinese except for Han.

NYHA: Classification of New York Heart Association

CHD: Coronary Heart Disease; PVD: Peripheral Vascular Disease; ACEI: angiotensin-converting enzyme inhibitors; COPD: Chronic Obstructive Pulmonary Disease; CKD: Chronic Renal Dysfunction; SD: Pulse pressure difference; LVEF: Left Ventricular Ejection Fraction; LVEDD: Left Ventricular end-diastolic diameter; WBC: white blood cells; ALT: alanine aminotransferase, AST: alkaline phosphatase; ALP: Alkaline phosphatase ;GGT: glutamyl transpeptidase; BUN :urea nitrogen; TP: total protein; PT: prothrombin time; ALB: Albumin; NT-proBNP: In (N-terminal brain sodium peptide); Hs-CRP: High-sensitivity C-reactive protein;

CPB- HGB: the hemoglobin of cardiopulmonary bypass.

CABG: cardiac artery bypass grafting

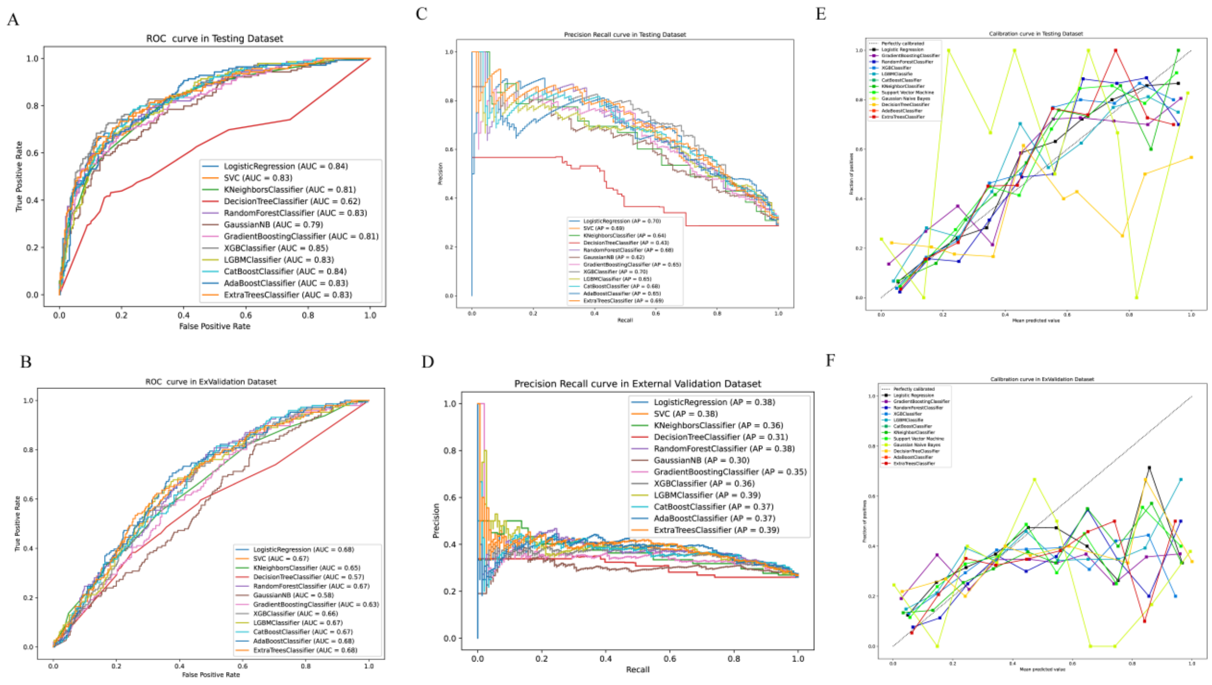

Figure S2:(A) The AUC of the machine learning models with surgical variables in the testing dataset

(B) The AUC of the machine learning models with surgical variables in the validation dataset

(C) The Precision recall curve of the machine learning models with surgical variables in the testing dataset

(D) The Precision recall curve in the machine learning models with surgical variables in the validation dataset

(E) The Calibration curve of the machine learning models with surgical variables in the testing dataset

(F) The Calibration curve in the machine learning models with surgical variables in the validation dataset

Supplementary Table S1: The accuracy, precision, recall, F1 score, and log loss in the development and external validation datasets.

Classifier	Accuracy			Precision			Recall			F1			Log loss		
	Test	ExValidation	MIMIC-IV	Test	ExValidation	MIMIC-IV	Test	ExValidation	MIMIC-IV	Test	ExValidation	MIMIC-IV	Test	ExValidation	MIMIC-IV
Logistic Regression	0.8079	0.7206	0.7541	0.7805	0.4304	0.6180	0.4604	0.2329	0.4468	0.5792	0.3022	0.5186	0.4406	0.6419	0.6400
Support Vector Machine	0.7128	0.7402	0.7034	0.0000	0.0000	0.0000	0.0000	0.0000	0.0000	0.0000	0.0000	0.0000	0.4582	0.6710	0.5469
KNeighborsClassifier	0.7583	0.7349	0.7390	0.7619	0.4483	0.8655	0.2302	0.0890	0.1419	0.3536	0.1486	0.2438	0.5429	0.7318	0.7378
DecisionTreeClassifier	0.6921	0.6566	0.6349	0.4569	0.3716	0.3842	0.3813	0.4658	0.3835	0.4157	0.4134	0.3839	10.6329	11.8613	12.6097
RamdomForestClassifier	0.7500	0.7206	0.7475	0.7813	0.3103	0.8708	0.1799	0.0616	0.1745	0.2924	0.1029	0.2907	0.4848	0.5446	0.5269
GaussianNB	0.7459	0.7171	0.7376	0.8077	0.3725	0.5955	0.1511	0.1301	0.3586	0.2545	0.1929	0.4476	2.9644	4.1371	5.0007
GradientBoostingClassifier	0.7769	0.7064	0.7333	0.7013	0.4059	0.5710	0.3885	0.2808	0.4046	0.5000	0.3320	0.4736	0.4959	0.7087	0.5643
XGBClassifier	0.7831	0.7100	0.7563	0.7361	0.3836	0.6921	0.3813	0.1918	0.3212	0.5024	0.2557	0.4388	0.4444	0.6015	0.5206
LGBMClassifier	0.7893	0.7100	0.7410	0.7229	0.4000	0.5943	0.4317	0.2329	0.3988	0.5405	0.2944	0.4773	0.6946	1.1730	0.9248
CatboostClassifier	0.7955	0.7224	0.7671	0.8030	0.4286	0.7500	0.3813	0.2055	0.3221	0.5171	0.2778	0.4507	0.4473	0.5681	0.5029
AdaBoostClassifier	0.7955	0.7224	0.7478	0.7778	0.4167	0.7653	0.4029	0.1712	0.2157	0.5308	0.2427	0.3366	0.6641	0.6700	0.6597

ExtraTreeClassifier	0.7686	0.7367	0.7541	0.8140	0.4444	0.7909	0.2518	0.0548	0.2320	0.3846	0.0976	0.3588	0.4729	0.5395	0.5332
